# The relationship between emotional eating disorders and problematic internet use in university students: the mediating role of mukbang behavior

**DOI:** 10.1590/1806-9282.20240343

**Published:** 2024-09-13

**Authors:** Nurten Elkin, Hasan Kütük, Deniz Mısra Gürol, Yağmur Dilan Bilgen

**Affiliations:** 1 Istanbul Gelisim University, Faculty of Health Science – İstanbul, Turkey.; 2 Istanbul Gelisim University, Vocational School of Health Services – İstanbul, Turkey.; 3 Istanbul Gelisim Unıversity, Faculty of Economics, Administrative And Social Sciences – İstanbul, Turkey.; 4 Istanbul Gelisim University – İstanbul, Turkey.

**Keywords:** Eating disorders, Addictive behavior, Addiction, Internet addiction

## Abstract

**OBJECTIVE:**

The aim of this study was to investigate the effect of watching mukbang on eating behavior and to emphasize its importance.

**METHODS:**

Students from various faculties at universities constitute the sample for this study. A total of 483 individuals participated in the study: 358 (74.1%) women and 125 (25.9%) men. The age range of the sample group varies between 18 and 50 years (M_age_=21.62; SD=3.85). In our study, the Emotional Eating Disorder Scale, the Mukbang Addiction Scale, and the Problematic Internet Use Scale were used. Reliability analysis, descriptive statistics, and correlational analysis of the data were carried out using IBM SPSS Statistics 24.

**RESULTS:**

There appears to be a positive relationship between emotional eating, mukbang addiction, and problematic internet use. A positive relationship was also found between emotional eating and mukbang addiction. It was found that mukbang addiction had a partial mediator role in the effect of problematic internet use on emotional eating.

**CONCLUSION:**

In the relationship between problematic internet use and emotional eating, mukbang addiction has played a mediating role. Therefore, when conducting a study between emotional eating and problematic internet use, it may be useful to examine the frequency of mukbang watching behavior in individuals. It can be crucial to include these people in educational programs to control problematic internet use or the habit of watching mukbang.

## INTRODUCTION

Nowadays, individuals use the Internet for a wide range of purposes, depending on their own needs. Some scholars have suggested that Internet overuse can be addictive and pathological; this idea is known more broadly as "technological addiction," which may include internet addiction, problematic internet use, addiction to digital games, problematic smartphone use, and so on^1^. Problematic internet use has become so common that many people have unintentionally become addicted to the internet over time, so now it is widely recognized as a disorder^2^. Problematic internet use is characterized by excessive use of the internet, emotional and mental health problems, as well as difficulties in education, employment, and social connections^3^. Addicts with problematic internet use (PIU) exhibit increased impulsivity and a loss of self-control in their actions^4^. These elements frequently result in unconscious internet use for the user. Moreover, PIU has been found to be linked with eating disorders, mental health issues, obesity, and a lack of physical activity. Negative feelings are a natural aspect of human existence. "Emotional eating" is the term for the phenomenon where some people eat to cope with negative emotions^5^. Research has indicated that anxiety and depression symptoms in certain situations lead to emotional eating in people^6,7^. Emotional eating may be the outcome of poor interoceptive awareness, alexithymia, inadequate emotion regulation techniques, and a confusion between internal sensations of hunger and satiety and physiological symptoms linked to emotions^5^.

As a mediator, mukbang refers to online broadcasts where people eat food and interact with the viewers. Mukbangers consume substantial amounts of food while interacting with viewers^7^. Although mukbang originated in South Korea 10 years ago, now it has spread to many other nations, where hundreds of thousands of people watch daily mukbang videos^8^. A study conducted to investigate the psychological characteristics of mukbang watchers and the psychological consequences of mukbang watching found that mukbang watching behavior has several underlying reasons, including social use, sexual use, entertainment use, escapist use, and eating use^9-12^.

One of the possible drawbacks of mukbang may be the ease with which people's standards of consumption might be influenced by those of others^13^. According to Donnar, mukbang may encourage unhealthy eating habits in viewers as well as mukbangers who are already dealing with various eating issues^9^. A study conducted with 140 emerging adults indicated that problematic mukbang watching was positively associated with both disordered eating and internet addiction^14^. The current study also found a favorable correlation between problematic mukbang watching and internet addiction. Choe indicates that by receiving both visual and auditory stimulation, viewers are able to experience the vicarious delight of eating the food they try to avoid from mukbang^10^. Furthermore, Bruno and Chung stressed that mukbang altered social and cultural food behavior by modifying viewers’ food and brand preferences, which may result in a decline in the cooking of homemade meals and an increase in the consumption of fast food^11^. Hong and Park claim that while extremely thin and slim mukbangers ate large portions of food without gaining weight, mukbang videos also have an impact on viewers’ perceptions of food intake and thinness^15^. This psychologically manipulates the viewers into doubting their attempts to maintain their physical health. As a result, problematic mukbang watching may encourage various eating disorders in different viewers.

Many psychologists have paid attention to the relationship between internet use and eating habits but there is very little attention to the effect of mukbang watching. This study aimed to highlight the importance of mukbang watching and its consequences in terms of eating behavior since there are no findings about mukbang, especially in Turkish literature.

## METHODS

### Ethical issues

Each stage of the study was conducted with careful consideration, according to the Declaration of Helsinki, and received ethical approval from the Istanbul Gelisim University Ethics Committee, with reference number 2023-06-92.

### Participants

Students from various faculties at universities constitute the sample for this study. A total of 483 individuals participated in the study: 358 (74.1%) women and 125 (25.9%) men. The age range of the sample group varies between 18 and 50 years (M_age_=21.62; SD=3.85). After examining the participants’ income levels, it was found that 35 (7.3%) people reported having low income, 330 (68.3%) reported as moderate income, and 118 (24.4%) reported as high income. The data were collected face-to-face by the research team from university students. Participants were informed about the study and the procedure so that they could withdraw from the study at any time. Informed consent was obtained from each participant before participation in the study, and participation was voluntary without receiving any payment.

### Data collection tools

The Emotional Eating Disorder Scale was developed by Garaulet et al. in 2012^16^. It includes a four-choice rating consisting of 10 items and three subscales. The Turkish version of the scale was adapted by Arslantaş et al.^17^, and the Cronbach alpha reliability coefficient was found to be 0.84. In this study, the Cronbach alpha reliability coefficient calculated for the scale was 0.86.

The Mukbang Addiction Scale is a 5-point Likert-type scale consisting of six items. It was developed by Kircaburun et al.^14^. In the original study, the Cronbach alpha coefficient was 0.95. In this study, the Cronbach alpha reliability coefficient calculated for the scale was 0.77.

The Problematic Internet Use Scale developed by Demetrovicset al.^18^ is a 5-point Likert-type scale consisting of six questions. As the score obtained from the scale increases, problematic use of the internet increases. An adaptation study into Turkish was conducted by Göktaş et al.^19^. In the original study, the Cronbach alpha coefficient was reported as 0.82. In this study, the Cronbach alpha coefficient calculated for the scale was 0.82.

### Statistical analyses

The steps listed below were followed while analyzing the data. Reliability analysis, descriptive statistics, and correlational analysis of the data were carried out using IBM SPSS Statistics 24. During the data analysis, skewness and kurtosis coefficients regarding normality and linearity assumptions were calculated. Since the skewness and kurtosis coefficients were between +1.96 and -1.96, it was determined that the data are normally distributed. Hypothesis testing was carried out using conditional process analysis. Hayes's^20^ PROCESS plugin was utilized for conditional process analysis. According to Hayes^20^, this analysis is a regression-based mediation model that allows for the inclusion of moderators. Regression-based mediation analysis, which is used to test theoretical models, is how this form of analysis is explained in the literature^21,22^.

Based on the relational screening model, this descriptive study uses the relational screening paradigm, which aims to determine the degree of difference between two or more variables and may include predictor, outcome, and mediator variables^23^. The predictor variable (X) is identified as problematic internet use, the mediator variable (M) as mukbang addiction, and the outcome variable (Y) as emotional eating in the model developed within the scope of this study.

## RESULTS

There appears to be a positive relationship between emotional eating (r=0.47), mukbang addiction (r=0.32), and problematic internet use. Additionally, a positive relationship (r=0.32) was found between emotional eating and mukbang addiction.

### First stage in mediation analysis

**Figure 1:**
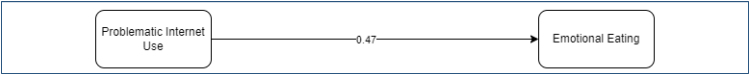
The model of problematic internet use to predict emotional eating.

The hypothesis to be tested in the mediation model states that problematic internet use and emotional eating are significantly correlated. The findings of the analysis carried out are given in Figure 1.

According to the findings, problematic internet use predicts emotional eating. By this regression model, problematic internet use explains 22% of the total variance [r^2^=0.22; F(1,481)=142.81; p≤0.01].

### Second stage in mediation analysis

**Figure 2:**
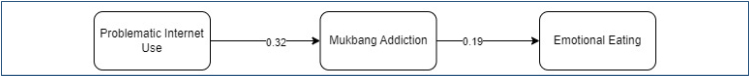
Model of how problematic internet use predicts mukbang addiction and how mukbang addiction predicts emotional eating.

According to the second assumption of the mediation analysis, problematic internet use should have a significant effect on mukbang addiction. The findings are given in Figure 2.

According to the results, problematic internet use is a positive predictor of mukbang addiction and explains 10% of the total variance [r^2^=0.10; F(1,481)=54.74; p≤0.01]. Another result of the analysis is that mukbang addiction is a positive predictor of emotional eating, and 10% of emotional eating is explained through mukbang addiction [r^2^=0.10; F(1,481)=56.44; p≤0.01].

### Third stage in mediation analysis

**Figure 3:**
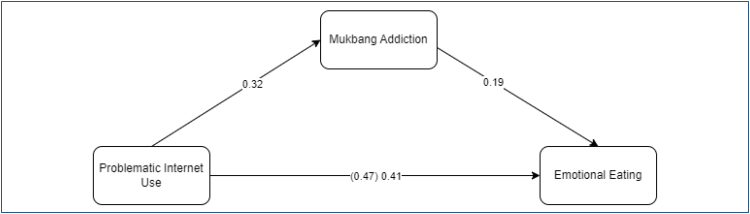
The mediating role of mukbang addiction in the relationship between problematic internet use and emotional eating.

In the last hypothesis regarding the analysis process, when mukbang addiction (M) is included as a mediating variable in the relationship between problematic internet use (X) and emotional eating (Y), it is expected that the power of problematic internet use on emotional eating will decrease or completely disappear. The results are given in Figure 3.

In this study, the coefficient of the relationship between problematic internet use and emotional eating was found to be 0.47. When mukbang addiction was added as a mediating variable to the relationship between these two variables, the relationship coefficient decreased to 0.41. The decrease in the correlation coefficient by 0.06 and the permanence of statistical significance show that mukbang addiction has a partial mediating role (c′=0.06; p≤0.01). This finding confirmed that mukbang addiction has a partial mediating role in the effect of problematic internet use on emotional eating, and the final hypothesis of the study was accepted. A bootstrap test was conducted to determine the significance of the partial mediating role of mukbang addiction. According to the results obtained from the bootstrap test, it was determined that there was no zero(0) value between the lower and upper limits in the 95% confidence interval (95%CI [0.03, 0.09]). This showed that the result of the bootstrap test was significant, that is, mukbang addiction had a partial mediating role in the relationship between problematic internet use and emotional eating.

## DISCUSSION

This study calls attention to the effect of mukbang watching behavior on the relationship between problematic internet use and emotional eating. According to a study done with Turkish university students, problematic internet use is a powerful predictor of eating attitudes^24^. A review of the literature reveals that the relationship between these concepts is indirect. The literature on this issue has made it clear that loneliness has a significant effect as well. A study examining the relationship between problematic internet use and loneliness found that those with higher scores in online addiction also reported feeling more alone^25^. This suggests that those who experience loneliness may use the internet more frequently, which may lead to emotional eating. These indirect findings in the literature suggest that problematic internet use is a predictor of emotional eating.

Another finding of this study is that mukbang addiction and problematic internet use are positively correlated. Research on the concept of mukbang is still incredibly scarce. In light of this, the findings from various studies were evaluated, and their significance for the link between problematic internet use and mukbang has been found to be indirect. A study indicates that there is a negative correlation between internet addiction and the level of enjoyment derived from interpersonal ties in one's daily life^25^. Hence, people utilize the internet more when they are less satisfied with their interpersonal interactions, and this might increase mukbang watching behavior as well.

The key finding of this study is that there is a significant positive relationship between emotional eating and mukbang addiction. Our results also align with the body of research demonstrating the relationship between emotional eating and mukbang watching. There are several reasons why people watch mukbang, such as the desire to escape from reality, feel less lonely while eating, and experience the vicarious joy of eating^14^. People who regularly watch mukbang, for instance, might consume more than they usually would because mukbangers routinely eat very large portions during their videos, and individuals can be influenced by others’ consumption^13^. It appears that some individuals have a desire to develop different eating habits to satisfy their emotional needs. The theory of this study is that these individuals may watch mukbang to relax themselves.

## CONCLUSION AND RECOMMENDATIONS

In the relationship between problematic internet use and emotional eating, mukbang addiction has played a mediating role. Therefore, when conducting a study between emotional eating and problematic internet use, it may be useful to examine the frequency of mukbang watching behavior in individuals. It can be crucial to include these people in educational programs to control problematic internet use or the habit of watching mukbang. It is believed that it would be useful to teach individuals alternative behaviors or habits that they can adopt in place of mukbang watching, and supporting people in this way by non-governmental organizations would be beneficial. Although many issues related to the digital world have been discussed in the existing literature, sufficient studies on mukbang watching have not been found. This study is believed to have the potential to contribute to this gap in the literature.

### Limitations

There are certain limitations to this study. One of them is that the data collected are cross-sectional. Since this study has a cross-sectional design, it would be more valuable to study longitudinal data to reveal the long-term results of the effect studied. The sample group for this study is limited to university students. It is known that problematic internet addiction levels among university students and adolescents are high. Therefore, a study conducted with adolescents could add a great deal to the body of literature. It is suggested that future research may pay attention to this issue.
